# World regional differences in outcomes for patients with peripheral artery disease: Insights from the EUCLID trial

**DOI:** 10.1177/1358863X211038620

**Published:** 2021-09-13

**Authors:** Lars Norgren, Rebecca North, Iris Baumgartner, Jeffrey S Berger, Juuso I Blomster, William R Hiatt, W Schuyler Jones, Brian G Katona, Kenneth W Mahaffey, Hillary Mulder, Manesh R Patel, Frank W Rockhold, F Gerry R Fowkes

**Affiliations:** 1 Faculty of Medicine and Health, Örebro University, Örebro, Sweden; 2 Department of Statistics, North Carolina State University, Raleigh, NC, USA; 3 Division of Angiology, Swiss Cardiovascular Centre, Inselspital, Bern University Hospital, University of Bern, Bern, Switzerland; 4 Departments of Medicine and Department of Surgery, New York University School of Medicine, New York, NY, USA; 5 Turku University Hospital, University of Turku, Turku, Finland; 6 Division of Cardiology, Department of Medicine, University of Colorado School of Medicine, Aurora, CO, USA; 7 Duke Clinical Research Institute, Durham, NC, USA; 8 Division of Cardiology, Department of Medicine, Duke University School of Medicine, Durham, NC, USA; 9 AstraZeneca Gaithersburg, Gaithersburg, MD, USA; 10 Stanford Center for Clinical Research, Stanford University School of Medicine, Stanford, CA, USA; 11 Usher Institute of Population Health Sciences and Informatics, University of Edinburgh, Edinburgh, UK

**Keywords:** epidemiology, geographical variation, outcome, peripheral artery disease (PAD), prevention, risk factors

## Abstract

Regional variations exist in the epidemiology of peripheral artery disease (PAD), in comorbidities, use of secondary prevention, and outcomes. Large studies of these variations in worldwide populations are rare. The EUCLID (Examining Use of tiCagreLor In peripheral artery Disease) trial included 13,885 patients with PAD from four geographical regions (Central/South America, Europe, Asia, North America) and compared monotherapy with ticagrelor and clopidogrel. Inclusion criteria were either an ankle–brachial index < 0.80 or a prior revascularization. The primary efficacy endpoint was time to first occurrence of any event in the composite of cardiovascular death, myocardial infarction, or ischemic stroke and did not differ between the study arms. This post hoc analysis of EUCLID confirmed that regional differences occurred in the inclusion criteria with more prior revascularization in North America (73.9%) and Asia (72.5%) compared with Central/South America (34.0%) and Europe (51.6%). The characteristics of patients also differed. Prior amputation at baseline was most frequent in Central/South America (6.3%) compared with other regions (1.6–2.8%). A history of stroke was most common in Asia, coronary heart disease in North America, and diabetes in Central/South America compared with other regions. The incidence of outcomes in patients with PAD varied by region. North America had the highest rate of the primary combined endpoint (5.97 events/100 patient-years). Corresponding rates were 4.80, 3.95, and 3.87 for Asia, Europe, and Central/South America, respectively. Hospitalization for acute limb ischemia (events/100 patient-years) was most frequent in Europe (0.75) and North America (0.74) compared with Asia (0.60) and Central/South America (0.33). Adjustment for inclusion criteria and relevant PAD characteristics did not have a major impact on these regional differences. Further adjustment for concomitant disease, risk factors, and preventive medication modified the regional differences only marginally. In conclusion, substantial regional differences were found in cardiovascular and limb outcomes in patients with PAD and were not explained by variation in the category of included patients, concomitant disease, risk factors, and prevention. Such differences, which may be due to variation in other factors such as background population rates or clinical care, need to be considered when designing and interpreting large international studies (**ClinicalTrials.gov Identifier: NCT01732822**).

## Introduction

The incidence of peripheral artery disease (PAD) is increasing worldwide, not least in low- and middle-income countries,^1^ with the greatest number of patients with PAD in the Southeast Asia and Western Pacific regions.^2^ Variation between populations and racial differences have been noted in the frequency of PAD, risk factors, and treatment. For example, in rural areas of the United States, amputation for PAD and concomitant diabetes mellitus have increased, specifically in African Americans and Native Americans.^3^ The REACH (REduction of Atherothrombosis for Continued Health) international registry, following patients with symptomatic PAD, showed at 4 years’ follow-up that the composite of cardiovascular death, myocardial infarction, or stroke was 17.6%, but with significantly lower rates in Japan compared with North America.^4^ Furthermore, Eastern Europe had a higher rate and Western Europe a lower rate of the composite endpoint compared with North America.^5^ In a review of ethnic differences,^6^ atherosclerotic PAD was found to be less prevalent in patients from Southeast Asia and those of African descent compared with white patients, despite a higher prevalence of diabetes mellitus in those from Southeast Asia.

Guidelines recommend prevention of cardiovascular disease as part of the treatment of patients with PAD.^7,8^ An international consensus panel performed a systematic review and concluded such prevention should be a high priority in the management of patients with PAD, particularly in low- and middle-income countries.^9^ Despite such recommendations, it is evident that antithrombotic treatment has been far from optimal, even following revascularization.^10^ The role of aspirin as the most frequently used drug has been questioned, and the slightly more effective drug, clopidogrel,^11^ has been used to varying extent. Importantly, in cardiovascular prevention studies, the PAD population has often been included only as a subgroup. The first large international study to include solely a PAD population was the EUCLID trial.

The EUCLID trial (Examining Use of tiCagreLor In peripheral artery Disease) was a prospective, multicenter, randomized, double-blind, event-driven study in 13,885 patients with symptomatic PAD from four regions (Central/South America, Europe, Asia, and North America) comparing monotherapy with ticagrelor and clopidogrel (ClinicalTrials.gov Identifier: NCT01732822).^12^ Patients were included with either an ankle–brachial index (ABI) < 0.80 or having had a prior revascularization. The primary efficacy endpoint was time to first occurrence of any event in the composite of cardiovascular death, myocardial infarction (MI), or ischemic stroke.^13^

Regional differences in the EUCLID population were recorded at baseline, including the proportion of patients recruited according to the two indications. Post hoc analyses of regional differences in the EUCLID trial have shown that hypertension was more common in White or Black/African American patients than in Asian or American Indian patients.^14^ It was also found that a prior MI recorded at baseline was more common in North America compared with the other regions.^15^ Regarding outcome, proportionally fewer patients from Central/South America compared with the other geographical regions suffered later acute limb ischemia.^16^ Major bleeding in the entire study cohort, as well as in cases following revascularization or amputation, was more frequent in North American participants compared with those in other regions.^17,18^

Given these differences, a further post hoc investigation of geographical variations was conducted. The aims of this analysis were to describe demographic differences in patients with PAD between the regions, and to determine if geographical region was associated with risk of major adverse cardiovascular events or limb outcomes, namely acute limb ischemia (ALI) requiring hospitalization or lower extremity revascularization (LER). In addition, factors that might be associated with these regional differences in outcomes, specifically concomitant cardiovascular disease, risk factors for cardiovascular disease, and preventive medication, were evaluated.

## Methods

### Study population

**Figure 1.**
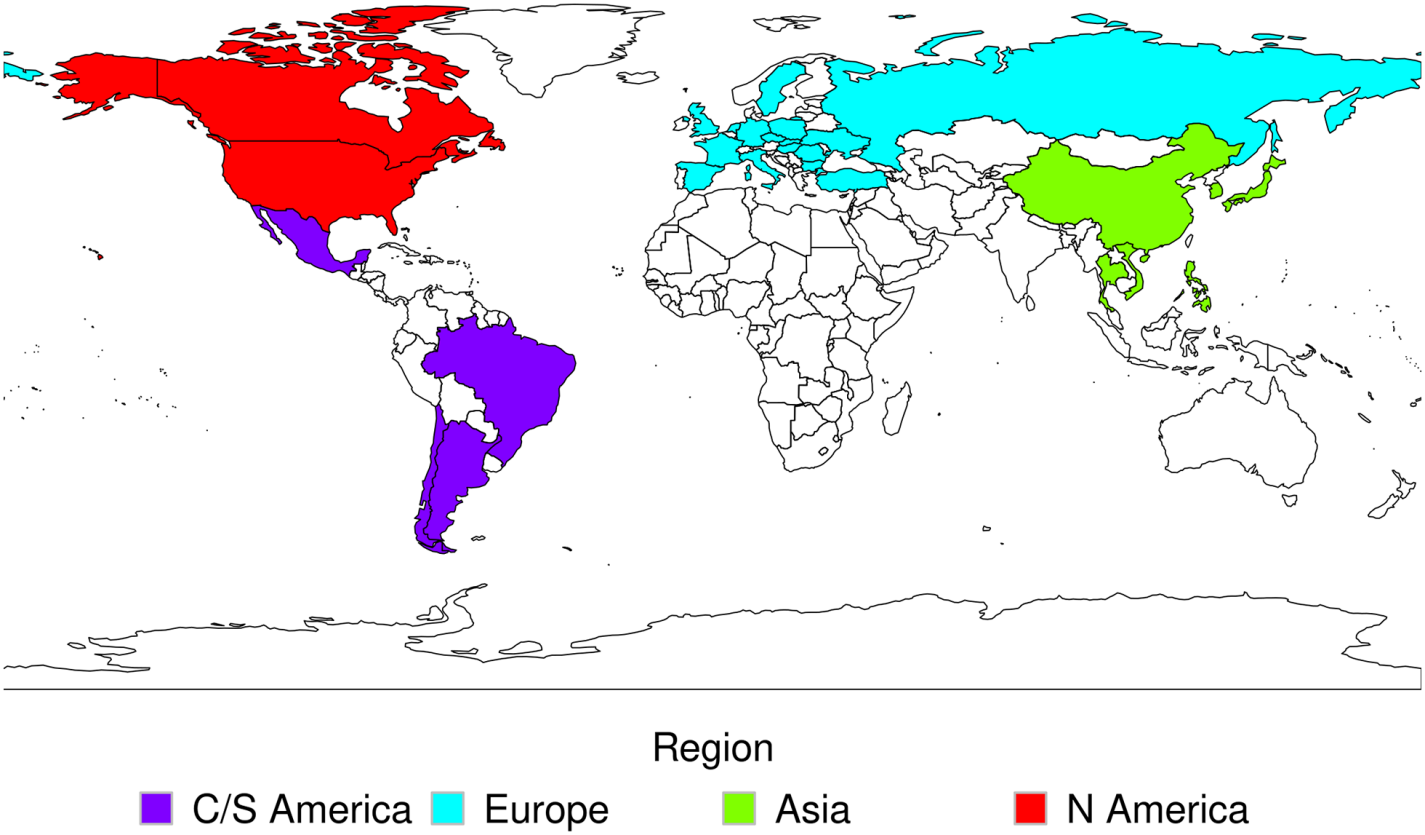
World map of countries in each region participating in EUCLID trial.

The EUCLID trial details and primary results have been previously published.^12,13^ Each patient provided written informed consent and the trial protocol was approved by ethics committees at participating sites. All 13,885 patients with PAD enrolled in the EUCLID trial were included in this analysis and grouped according to region. The study population comprised 1740 participants (12.5%) in Central/South America (Argentina, Brazil, Chile, Mexico), 1602 (11.5%) in Asia (China, Japan, Philippines, South Korea, Thailand, Vietnam), 3045 (21.9%) in North America (Canada, USA), and 7498 (54.0%) in Europe (Bulgaria, Czech Republic, France, Germany, Hungary, Italy, Netherlands, Poland, Romania, Russian Federation, Slovakia, Spain, Sweden, Turkey, Ukraine, United Kingdom). Regions are depicted in Figure 1.

### Endpoints

The primary endpoint considered is a composite of cardiovascular death, MI, or ischemic stroke, as in the original study. Occurrences of MI and ischemic stroke included both fatal and nonfatal events. All-cause death, ALI requiring hospitalization, LER, and the components of the composite endpoint were also studied.

### Statistical analysis

Baseline characteristics were stratified by region. Continuous variables are reported as medians with the 25th and 75th percentiles or means with SDs and were assessed with Kruskal–Wallis tests. Categorical variables are reported as counts and percentages and were assessed with chi-squared tests. Clinical outcomes were assessed by region: Central/South America, Asia, North America, and Europe (reference region). Incidence rates and number of events are reported for each outcome, and Cox proportional hazards models were fit to determine the association between region and outcomes. For all models, hazard ratios (HR), 95% CI, and *p*-values are reported. Both unadjusted and adjusted models were fit. Outcomes were adjusted for age, sex, inclusion criteria, and severity of disease, where severity of disease is defined as asymptomatic, all intermittent claudication, or all critical limb ischemia (CLI). The proportional hazards assumption – which assumes hazards only differ by a multiplicative constant for all individuals across regions – was assessed for region using Schoenfeld residuals. Owing to a violation of the proportional hazards assumption for the outcome of MI and region of Asia, MI events in Asia were partitioned into those that occurred in the first year or those that occurred after the first year and modeled separately for each time interval. Kaplan–Meier curves stratified by region are also presented. A sensitivity analysis was conducted in which the European region was split into Eastern and Western Europe, and North America was the reference region.

Additional Cox models were fit for the primary composite outcome, for all-cause death, and for LER to investigate the relationships between outcomes and three groups of factors: concomitant cardiovascular disease, risk factors for cardiovascular disease, and preventive medications. Concomitant cardiovascular disease includes prior occurrence of stroke, carotid stenosis or carotid revascularization, MI, percutaneous coronary intervention (PCI), and coronary artery bypass graft surgery (CABG). Risk factors for cardiovascular disease include diabetes, current smoking, hypertension, and hyperlipidemia. Preventive medications include baseline use of statins, angiotensin receptor blockers, and angiotensin-converting enzyme (ACE) inhibitors. Initially, a base model was fit that adjusted for age, sex, inclusion criteria, and severity of disease. Each group of factors was sequentially added to the base model, and the corresponding HRs, 95% CIs, and *p*-values are reported. The base model with concomitant cardiovascular disease was denoted as Model 1, Model 1 plus risk factors for cardiovascular disease as Model 2, and Model 2 plus preventive medication as Model 3. As a sensitivity analysis, forward stepwise regression models that included all three groups of factors were also fit, forcing the base model factors into the model. The selected factors, HRs, 95% CIs, and *p*-values are reported for each outcome. All analyses were conducted with SAS software, Version 9.4 (SAS Institute, Inc., Cary, NC, USA).

## Results

### Baseline characteristics

The baseline characteristics of the participants are shown in Table 1. In each region, fewer participants were female than male, with relatively fewer females in Asia (20.6%) and Europe (24.4%) compared with North America (35.0%) and Central/South America (38.2%). The median weight of participants was lowest in Asia (62 kg). Relatively more participants were included in the trial on the grounds of prior revascularization in North America (73.9%) and Asia (72.5%) than in Europe (51.6%) and Central/South America (34.0%). The distribution of presenting limb symptoms was comparable between the regions, except that the proportion who were asymptomatic was particularly high in Asia (34.5%) and low in Central/South America (7.4%). Of the asymptomatic patients, 95% had a prior revascularization (data not shown). CLI, defined as rest pain and/or minor or major tissue loss, was present in a low proportion of patients (4.6%), especially in Europe and North America compared with Asia and Central/South America. Although major amputation at baseline was rare, a higher proportion was recorded for Central/South America (6.3%) compared with the other regions (1.6–2.8%).

**Table 1.** Baseline characteristics by region.

Characteristic	Central/South America (*n* = 1740)	Europe (*n* = 7498)	Asia (*n* = 1602)	North America (*n* = 3045)	*p*-value
Age, median (25th, 75th), years	67 (61, 74)	65 (59, 71)	70 (63, 75)	67 (61, 73)	< 0.001
Female sex, no. (%)	664 (38.2%)	1828 (24.4%)	330 (20.6%)	1066 (35.0%)	< 0.001
Weight, median (25th, 75th), kg	74 (65, 84)	79 (70, 89)	62 (54, 70)	82 (70, 94)	< 0.001
Inclusion criteria for randomization					< 0.001
Previous revascularization, no. (%)	591 (34.0%)	3872 (51.6%)	1162 (72.5%)	2250 (73.9%)	
ABI value, mean (SD)	0.70 (0.23)	0.76 (0.23)	0.81 (0.25)	0.82 (0.21)	< 0.001
ABI or TBI criteria, no. (%)	1149 (66.0%)	3626 (48.4%)	440 (27.5%)	795 (26.1%)	
ABI value, mean (SD)	0.66 (0.20)	0.62 (0.14)	0.62 (0.15)	0.64 (0.12)	< 0.001
TBI value, mean (SD)	0.57 (0.22)	0.49 (0.22)	0.46 (0.12)	0.44 (0.16)	0.007
Limb symptoms, no. (%)					< 0.001
Asymptomatic	128 (7.4%)	1311 (17.5%)	553 (34.5%)	609 (20.0%)	
Mild or moderate claudication	998 (57.4%)	4053 (54.1%)	730 (45.6%)	1629 (53.5%)	
Severe claudication	508 (29.2%)	1818 (24.3%)	212 (13.2%)	690 (22.7%)	
Pain while at rest	51 (2.9%)	188 (2.5%)	42 (2.6%)	97 (3.2%)	
Minor tissue loss	44 (2.5%)	99 (1.3%)	48 (3.0%)	16 (0.5%)	
Major tissue loss	11 (0.6%)	26 (0.3%)	17 (1.1%)	4 (0.1%)	
Major amputation above the ankle	108 (6.3%)	137 (1.8%)	45 (2.8%)	49 (1.6%)	< 0.001
Minor amputation	175 (10.1%)	272 (3.6%)	82 (5.1%)	76 (2.5%)	< 0.001
Medical history, no. (%)					
Stroke	126 (7.2%)	550 (7.3%)	258 (16.1%)	209 (6.9%)	< 0.001
TIA	42 (2.4%)	212 (2.8%)	62 (3.9%)	191 (6.3%)	< 0.001
CAD	449 (25.8%)	1838 (24.5%)	308 (19.2%)	1437 (47.2%)	< 0.001
MI	319 (18.3%)	1283 (17.1%)	158 (9.9%)	762 (25.0%)	< 0.001
Carotid stenosis or carotid revascularization	122 (7.4%)	1326 (18.7%)	182 (13.2%)	897 (29.7%)	< 0.001
Diabetes mellitus type I or II	989 (56.8%)	2447 (32.6%)	689 (43.0%)	1220 (40.1%)	< 0.001
Hypertension	1350 (77.6%)	5757 (76.8%)	1123 (70.1%)	2627 (86.3%)	< 0.001
Hyperlipidemia	1232 (70.8%)	5551 (74.1%)	883 (55.1%)	2814 (92.4%)	< 0.001
Tobacco use, no. (%)					< 0.001
Current	385 (22.1%)	2502 (33.7%)	373 (23.3%)	1029 (33.8%)	
Former	801 (46.0%)	3221 (43.4%)	844 (52.7%)	1664 (54.6%)	
Never	554 (31.8%)	1693 (22.8%)	385 (24.0%)	352 (11.6%)	
Medication use before randomization, no. (%)					
Aspirin	1089 (62.6%)	4950 (66.0%)	905 (56.5%)	2327 (76.4%)	< 0.001
Clopidogrel	217 (12.5%)	2166 (28.9%)	551 (34.4%)	1539 (50.5%)	< 0.001
Statin	1134 (65.2%)	5463 (72.9%)	1012 (63.2%)	2572 (84.5%)	< 0.001
ACE inhibitor	706 (40.6%)	3343 (44.6%)	205 (12.8%)	1381 (45.4%)	< 0.001
ARB	526 (30.2%)	1654 (22.1%)	583 (36.4%)	725 (23.8%)	< 0.001
Cilostazol	691 (39.7%)	252 (3.4%)	760 (47.4%)	392 (12.9%)	< 0.001

ABI, ankle–brachial index; ACE, angiotensin-converting enzyme; ARB, angiotensin receptor blocker; CAD, coronary artery disease; MI, myocardial infarction; TBI, toe–brachial index; TIA, transient ischemic attack.

Table 1 also shows that histories of concomitant medical conditions varied between the regions. A history of stroke occurred more commonly in Asia (16.1%) whereas coronary heart disease and MI were most common in North America (47.2% and 25.0%, respectively). Diabetes mellitus was particularly common in Central/South America (56.8%). Hypertension was frequent in all regions, affecting over 70% of participants. Hyperlipidemia was extremely common in North America, affecting 92.4% of participants. Tobacco use was high in all regions, with at least two-thirds of participants having a history of current or former smoking. Prior to entry into the trial, secondary preventive medications were taken frequently. These included statins (slightly more so in North America and Europe), ACE inhibitors/angiotensin receptor blockers (ACE inhibitors less frequently in Asia), and antiplatelet medication. Cilostazol was most frequently used in Asia (47.4%), but very rarely in Europe (3.4%).

### Regional clinical outcomes

**Figure 2.**
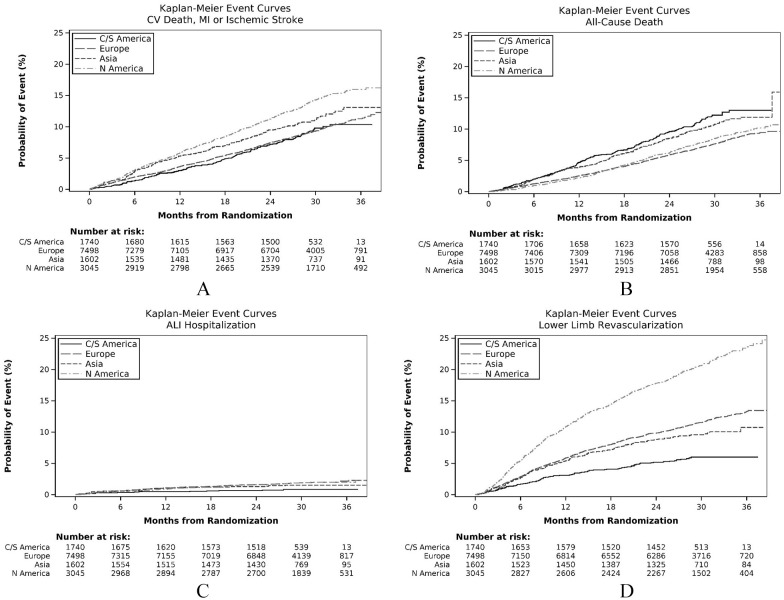
Kaplan–Meier event curves for clinical outcomes. (A) Composite endpoint (cardiovascular death, MI, or stroke); (B) all-cause death; (C) ALI hospitalization; (D) lower extremity revascularization. ALI, acute limb ischemia; C/S America, Central/South America; CV, cardiovascular; MI, myocardial infarction.

The incidence rates of the clinical outcomes in each region and the unadjusted HRs to Europe are shown in Table 2; the cumulative probabilities of an event during follow-up are shown in Figures 2A–D. North America had the highest probability of the combined endpoint of cardiovascular death, MI, or stroke (Figure 2A). For the individual outcomes, the probability of cardiovascular death was particularly high in Central/South America (Figure S1) and lowest in North America, whereas MI was most common in North America and low in Central/South America (Figure S2). The rate of ischemic stroke was highest in Asia and lowest in Central/South America (Figure S3). All-cause death had a similar regional distribution to cardiovascular death, being highest in Central/South America and lowest in Europe and North America (Figure 2B). For limb outcomes, the probability of hospitalization for ALI was highest in Europe and North America and lowest in Central/South America (Figure 2C). The probability of LER was similar to hospitalization for ALI, except that the probability of revascularization was much higher in North America compared with the other regions (Figure 2D).

**Table 2.** Unadjusted associations between region and clinical outcomes.

Clinical outcome	Central/South America			Asia			North America			Europe	Global *p*-value^a^
	Incidence rate (*n*)	HR (95% CI)	*p*-value	Incidence rate (*n*)	HR (95% CI)	*p*-value	Incidence rate (*n*)	HR (95% CI)	*p*-value	Incidence rate (*n*)	
CV death/MI/stroke	3.87 (151)	0.98 (0.82–1.17)	0.813	4.80 (180)	1.21 (1.03–1.43)	0.021	5.97 (436)	1.51 (1.34–1.71)	< 0.001	3.95 (724)	< 0.001
CV death	2.88 (114)	1.51 (1.23–1.87)	< 0.001	2.23 (87)	1.16 (0.92–1.47)	0.206	1.85 (143)	0.96 (0.79–1.17)	0.701	1.92 (362)	< 0.001
MI	0.66 (26)	0.42 (0.28–0.62)	< 0.001	1.87 (71)	1.71 (1.21–2.40) 0.82 (0.56–1.20)	0.002 0.299	3.96 (292)	2.50 (2.13–2.94)	< 0.001	1.59 (294)	< 0.001
Stroke	0.48 (19)	0.59 (0.36–0.95)	0.029	1.44 (55)	1.75 (1.29–2.38)	< 0.001	0.97 (74)	1.20 (0.91–1.58)	0.207	0.82 (152)	< 0.001
All-cause death	5.05 (203)	1.60 (1.36–1.87)	< 0.001	4.49 (176)	1.41 (1.19–1.66)	< 0.001	3.51 (277)	1.10 (0.95–1.27)	0.198	3.20 (607)	< 0.001
ALI	0.33 (13)	0.43 (0.24–0.76)	0.003	0.60 (23)	0.79 (0.51–1.22)	0.283	0.74 (56)	0.98 (0.72–1.34)	0.899	0.75 (140)	0.025
LER	2.49 (95)	0.50 (0.40–0.62)	< 0.001	4.10 (150)	0.83 (0.70–0.99)	0.033	9.33 (631)	1.89 (1.71–2.10)	< 0.001	4.92 (862)	< 0.001

Reference is Europe. HR (95% CI) and *p*-values for MI in Asia correspond to time intervals (0, 365) and (365, . . .) days, respectively.

Incidence rate: number of events (*n*) per 100 patient-years.

a Global *p*-value from the overall association test.

ALI, acute limb ischemia; CV, cardiovascular; HR, hazard ratio; LER, lower extremity revascularization; MI, myocardial infarction.

### Adjustment of regional differences on outcomes

HRs for the different clinical outcomes for each region with reference to Europe are shown in Table 3, with adjustment for age, sex, severity of PAD, and inclusion criteria for the trial. The differences between the regions were very similar to those observed for the unadjusted associations (Table 2), with the HRs being slightly lower than the unadjusted HRs, with the exception of LER in Central/South America. This suggests that regional differences in the types of patients with PAD recruited (as defined by the additional covariates above) did not have a major influence on clinical outcomes.

**Table 3.** Adjusted associations between region and clinical outcomes.

Clinical outcome	Central/South America		Asia		North America		Global *p*-value*
	HR (95% CI)	*p*-value	HR (95% CI)	*p*-value	HR (95% CI)	*p*-value	
CV death/MI/stroke	0.92 (0.77–1.10)	0.345	1.05 (0.89–1.24)	0.553	1.41 (1.24–1.59)	< 0.001	< 0.001
Model 1	0.92 (0.77–1.10)	0.345	1.07 (0.90–1.27)	0.429	1.24 (1.09–1.42)	< 0.001	0.003
Model 2	0.86 (0.72–1.03)	0.094	1.00 (0.84–1.18)	0.973	1.25 (1.10–1.43)	< 0.001	< 0.001
Model 3	0.85 (0.71–1.02)	0.079	0.99 (0.83–1.18)	0.942	1.24 (1.08–1.41)	0.002	< 0.001
CV death	1.34 (1.08–1.66)	0.008	1.00 (0.79–1.28)	0.982	0.90 (0.74–1.10)	0.312	0.018
MI	0.41 (0.27–0.61)	< 0.001	1.45 (1.03–2.05) 0.69 (0.47–1.02)	0.034 0.063	2.23 (1.89–2.64)	< 0.001	< 0.001
Stroke	0.55 (0.34–0.90)	0.016	1.56 (1.13–2.15)	0.006	1.16 (0.87–1.55)	0.300	0.001
All-cause death	1.46 (1.25–1.72)	< 0.001	1.16 (0.98–1.38)	0.090	1.00 (0.86–1.15)	0.970	< 0.001
Model 1	1.47 (1.25–1.73)	< 0.001	1.18 (0.99–1.41)	0.062	0.96 (0.83–1.12)	0.640	< 0.001
Model 2	1.43 (1.21–1.68)	< 0.001	1.10 (0.92–1.31)	0.302	1.01 (0.86–1.18)	0.937	< 0.001
Model 3	1.43 (1.21–1.68)	< 0.001	1.13 (0.94–1.36)	0.186	0.99 (0.85–1.16)	0.892	< 0.001
ALI	0.53 (0.30–0.95)	0.032	0.67 (0.43–1.05)	0.083	0.78 (0.57–1.07)	0.122	0.050
LER	0.57 (0.46–0.71)	< 0.001	0.74 (0.62–0.88)	< 0.001	1.60 (1.44–1.78)	< 0.001	< 0.001
Model 1	0.57 (0.46–0.71)	< 0.001	0.73 (0.61–0.87)	< 0.001	1.55 (1.39–1.73)	< 0.001	< 0.001
Model 2	0.56 (0.46–0.70)	< 0.001	0.74 (0.62–0.89)	0.001	1.48 (1.32–1.66)	< 0.001	< 0.001
Model 3	0.56 (0.45–0.70)	< 0.001	0.75 (0.63–0.90)	0.002	1.49 (1.33–1.66)	< 0.001	< 0.001

Reference is Europe (Table 2). All outcomes adjusted for age, sex, inclusion criteria, and severity of disease. HR (95% CI) and *p*-values for MI in Asia correspond to time intervals (0, 365) and (365, . . .) days, respectively.

Model 1 adjusts for concomitant cardiovascular diseases (prior stroke, carotid stenosis or revascularization, MI, PCI, CABG).

Model 2 adds cardiovascular disease risk factors to Model 1 (diabetes, smoking, hypertension, hyperlipidemia).

Model 3 adds preventive medications to Model 2 (statins, angiotensin receptor blockers, ACE inhibitors).

* Global *p*-value from the overall association test.

ACE, angiotensin-converting enzyme; ALI, acute limb ischemia; CABG, coronary artery bypass graft surgery; CV, cardiovascular; HR, hazard ratio; LER, lower extremity revascularization; MI, myocardial infarction; PCI, percutaneous coronary intervention.

These findings were further analyzed by sequential adjustment of the HRs for regional differences by concomitant cardiovascular disease, risk factors, and preventive medications (Table 3). The main effects of adjusting for concomitant cardiovascular disease (Model 1 compared with the base model) were found in North America, where the HRs were reduced but with considerable overlap of 95% CIs for the combined clinical endpoint of cardiovascular death, MI, or stroke (HR 1.41; 95% CI 1.24–1.59 to 1.24; 95% CI 1.09–1.42), for all-cause death (HR 1.00; 95% CI 0.86–1.15 to 0.96; 95% CI 0.83–1.12), and for LER (HR 1.60; 95% CI 1.44–1.78 to 1.55; 95% CI 1.39–1.73). Further adjustment for risk factors (diabetes, smoking, hypertension, hyperlipidemia) led to reductions (Model 2 compared with Model 1) in HRs with overlapping 95% CIs in Central/South America and in Asia for both the combined endpoint of cardiovascular death, MI, or stroke, and for all-cause death. On the other hand, in North America, the HR for all-cause death increased slightly (HR 0.96; 95% CI 0.83–1.12 to 1.01; 95% CI 0.86–1.18). The final further adjustment for preventive medications (Model 3 compared with Model 2) had minimal impact on the HRs. Overall, the HRs for Model 3 compared with the base model across the regions indicate that the adjustments for concomitant cardiovascular disease, risk factors, and preventive medications had only a slight impact on the magnitude of the HRs. This was further demonstrated in the stepwise adjusted associations (Table S1) in which the final adjusted HRs were very similar to those in Model 3 for the sequentially adjusted associations (Table 3).

### Sensitivity analysis

In the sensitivity analysis in which Europe was divided into Western and Eastern regions, the main difference in baseline characteristics between the two regions was that previous revascularization was a more common inclusion criterion in Western than Eastern Europe (63.8% vs 46.0% of trial participants, respectively) (Table S2). In comparing unadjusted and adjusted associations between region and clinical outcomes with North America as the reference region, the HRs for cardiovascular death were higher in Eastern than Western Europe and the HRs for LER were higher in Western than Eastern Europe (Tables S3 and S4). The Kaplan–Meier curves (Figures S4 to S10) show that Western Europe had the lowest rates of cardiovascular and all-cause deaths and, along with North America, had considerably higher rates of LER compared to the other regions.

## Discussion

### EUCLID trial

EUCLID, the largest trial conducted in patients with PAD to date, included participants from 28 countries in four regions. The indications for inclusion were wide, ranging from mild or moderate claudication to CLI. Mild or moderate claudication dominated, and the patients with CLI had a relatively mild form of this serious stage of PAD.^19^ Thus, unsurprisingly, the 1-year mortality for patients with CLI was comparatively low (8.9%). Patients with a prior revascularization were also included, even if asymptomatic. The primary efficacy endpoint of the composite of cardiovascular death, MI, or ischemic stroke was reached by a relatively small proportion of patients (10.7%), with no difference between the ticagrelor and clopidogrel groups. This homogeneity meant that the two groups could be combined to study the whole PAD population. Given very limited previous information on global differences between regions on outcomes of PAD, and despite the relatively low outcome rates, further investigation was of interest, particularly given the very large number of patients with PAD in the EUCLID trial.

### Factors influencing outcomes

It is important to note that patients were selected by the investigators based on standard inclusion criteria. Whether differences between regions in the patients with PAD reflect primarily epidemiological variation or differences in selection remains difficult to conclude, even though adjustment was made in the analysis for age, sex, inclusion criteria, and severity of PAD. Selection may have been influenced by other factors such as availability of resources and the investigators’ interest and competence. It is also reasonable to assume that interventionists and vascular surgeons may have included more patients based on a prior revascularization whereas noninterventionists may have included more patients based on the ABI criteria. Furthermore, the type and quality of care provided by different investigators may have influenced outcomes.

In Central/South America, one-third of the patients were included after a prior revascularization, whereas in both Asia and North America more than two-thirds were included based on that indication. This difference may explain the lower rate of asymptomatic patients in Central/South America. This region also had the largest proportion of patients with diabetes. It may be hypothesized that the higher rate of major amputation in this region was caused by more diabetes and fewer revascularizations. On the other hand, in regions where prior revascularization was the dominant inclusion criterion, lower limb outcomes (revascularization, ALI, and hospitalization) might be expected to be more common, as recorded for North America and to some extent Europe.

Antiplatelet medication as part of secondary prevention, mainly aspirin, was used by a majority of patients in all regions. In those with a high usage of both aspirin and clopidogrel (specifically North America), dual therapy may have been used more frequently compared with other regions. One reason might be a greater proportion of endovascular procedures in which dual antiplatelet treatment is regularly considered. The variation of cilostazol use was extremely wide: only 3.4% in Europe but 47.4% in Asia. A likely explanation is that the European Medical Association recommended restricted use of cilostazol in 2013^20^ and a meta-analysis concluded cilostazol was ineffective in preventing major adverse cardiovascular events in patients with PAD.^21^

### Regional differences in outcomes

The association between region and clinical outcomes showed that the highest probability of the combined endpoint, cardiovascular death, MI, or stroke, for the 36 months of follow-up, occurred in North America. The highest probability of MI was also recorded for this region, whereas cardiovascular death was most common in Central/South America and lowest in North America. In Asia, the greatest risk for stroke was found. Central/South America also had the highest probability for all-cause death. LER and hospitalization for ALI were most common in Europe and North America.

Adjustment for the fundamental characteristics of patients with PAD (age, sex, inclusion criteria, and severity of disease) did not modify the regional differences in outcomes to any great extent. Adding sequential adjustment for concomitant cardiovascular disease, risk factors for cardiovascular disease, and preventive medication modified the risk of MI in North America and the risk of cardiovascular death in Central/South America only slightly.

### Study limitations

The lack of explanation for these regional differences in outcomes could be due to some limitations in the scope of the analysis. The concomitant diseases, risk factors, and preventive medications may not have been sufficiently comprehensive to identify those contributing in a major way to the regional differences. Also, other possible major influences were not studied, such as the types of specialists involved in managing patients, the quality and scope of clinical care, and the genetics and ethnicity of the included patients. Furthermore, the EUCLID population included relatively mild stages of PAD and whether more pronounced regional variations might have been revealed in more severely diseased patients could not be verified. Importantly, the overall background event rates in the population as a whole may have had a dominant effect on the event rate in the trial population rather than the specific disease under study or the treatments administered.

## Conclusion

In conclusion, in the EUCLID trial, differences in cardiovascular and lower limb outcomes were found between patients with PAD in different world regions. These differences were not fully explained by different categories of patients with PAD recruited into the trial nor by differences in concomitant diseases, cardiovascular risk factors, or preventive medications. These regional differences in outcomes can influence the overall results in large international trials and, if feasible, should be understood and assessed when designing such studies. Factors other than the trial treatment which might influence regional differences in outcome should also be considered. Furthermore, a particularly high rate of a specific outcome in one region, especially when combined with a high proportion of trial participants recruited from that region, might affect the overall results of the trial that are typically based on the total population and a composite outcome.

## Supplemental Material

sj-pdf-1-vmj-10.1177_1358863X211038620 – Supplemental material for World regional differences in outcomes for patients with peripheral artery disease: Insights from the EUCLID trial Click here for additional data file. Supplemental material, sj-pdf-1-vmj-10.1177_1358863X211038620 for World regional differences in outcomes for patients with peripheral artery disease: Insights from the EUCLID trial by Lars Norgren, Rebecca North, Iris Baumgartner, Jeffrey S Berger, Juuso I Blomster, William R Hiatt, W Schuyler Jones, Brian G Katona, Kenneth W Mahaffey, Hillary Mulder, Manesh R Patel, Frank W Rockhold and F Gerry R Fowkes in Vascular Medicine
