# Supplementary material for: World regional differences in outcomes for patients with peripheral artery disease: Insights from the EUCLID trial
Source: Vasc Med. 2021 Sep 13;27(1):21–9. doi: 10.1177/1358863X211038620 (PMC8808360; doi:10.1177/1358863X211038620)
Supplement: sj-pdf-1-vmj-10.1177_1358863X211038620 – Supplemental material for World regional differences in outcomes for patients with peripheral artery disease: Insights from the EUCLID trial [file sj-pdf-1-vmj-10.1177_1358863X211038620.pdf]

Supplement for World Regional Differences in Outcomes for Patients with Peripheral Artery Disease: EUCLID Trial

Table S1. Stepwise adjusted associations between region and clinical outcomes

	Central/South America		Asia		North America		
Clinical Outcome	HR (95% CI)	P-value	HR (95% CI)	P-value	HR (95% CI)	P-value	Global P-value*
CV death/MI/Stroke	0.85 (0.71–1.02)	0.077	1.01 (0.85–1.19)	0.951	1.28 (1.13–1.45)	<.001	<.001
All-cause death	1.43 (1.21–1.68)	<.001	1.14 (0.96–1.37)	0.145	0.99 (0.85–1.15)	0.864	<.001
LER	0.56 (0.45–0.70)	<.001	0.74 (0.62–0.88)	<.001	1.55 (1.40–1.73)	<.001	<.001

*P-value from the overall association test. Reference is Europe. All outcomes adjusted for age, sex, inclusion criteria, and severity of disease. CV death/MI/stroke adjusted for diabetes, smoking, statins, and prior MI, stroke, and carotid stenosis or revascularization. All-cause death adjusted for diabetes, smoking, hyperlipidemia, statins, angiotensin receptor blockers, and prior MI, stroke, and carotid stenosis or revascularization. Lower extremity revascularization adjusted for diabetes, smoking, and prior carotid stenosis or revascularization.
CI indicates confidence interval; CV, cardiovascular; HR, hazard ratio; LER, lower extremity revascularization; MI, myocardial infarction.

Figure S1. Kaplan Meier event curves for cardiovascular death

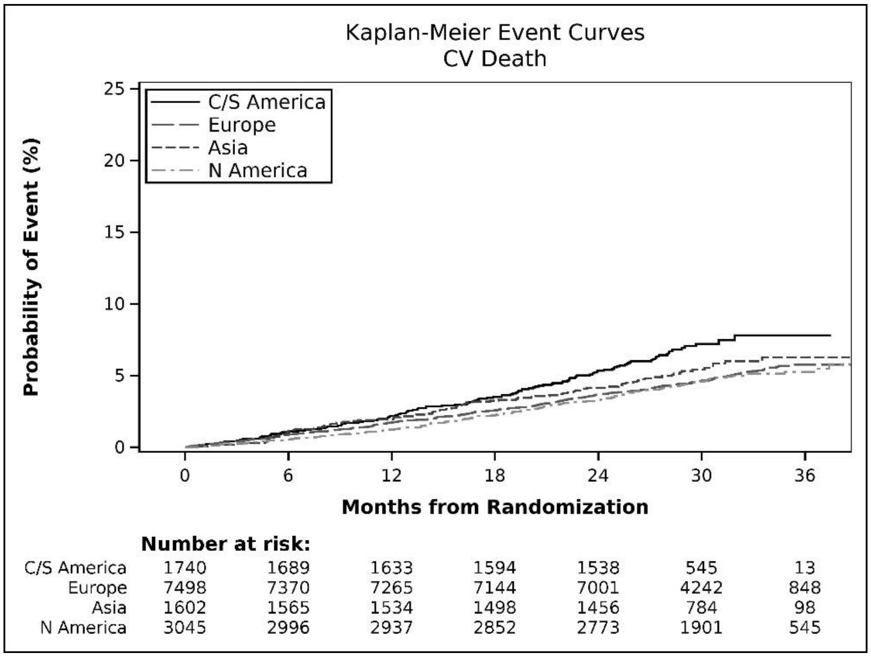

Figure S2. Kaplan Meier event curves for myocardial infarction (MI)

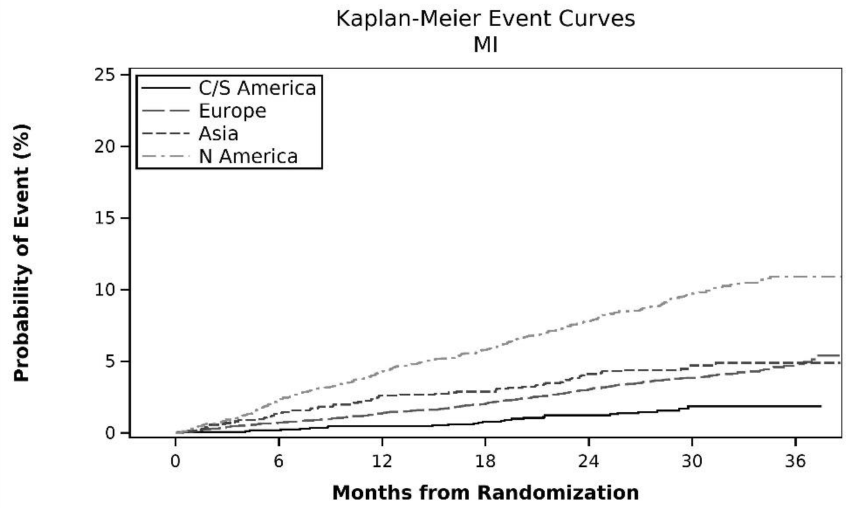

Number at risk:

	0	6	12	18	24	30	36
C/S America	1740	1680	1620	1572	1511	533	13
Europe	7498	7311	7144	6987	6785	4062	807
Asia	1602	1544	1497	1458	1402	756	93
N America	3045	2929	2815	2688	2570	1733	501

Figure S3. Kaplan Meier event curves for ischemic stroke

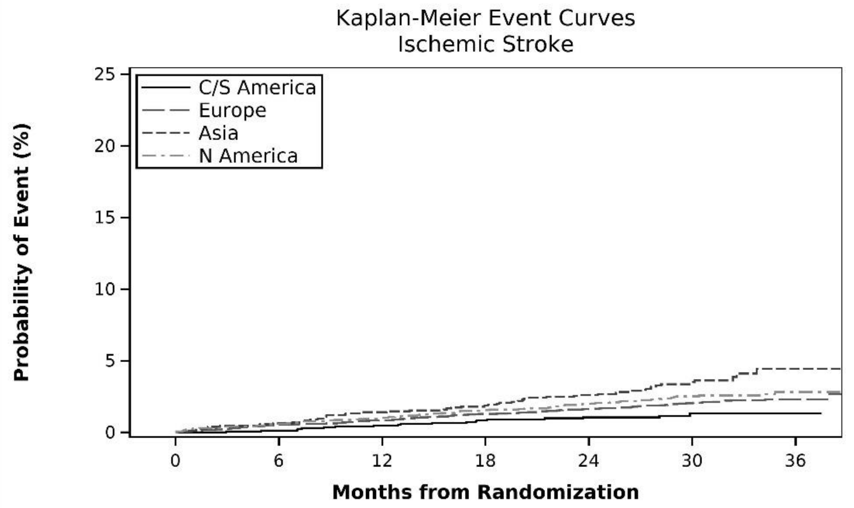

Number at risk:

	0	6	12	18	24	30	36
C/S America	1740	1680	1621	1571	1515	539	13
Europe	7498	7318	7174	7025	6854	4127	816
Asia	1602	1552	1512	1465	1411	760	95
N America	3045	2971	2894	2785	2695	1833	525

As a sensitivity analysis, the European region was divided into Western and Eastern Europe, which coincidentally coincides with the division based on the low- and high-risk classification for cardiovascular disease as specified by the 2016 European Guidelines on Cardiovascular Disease Prevention in Clinical Practice (Piepoli MF et al. European Heart Journal 2016;37:2315-81). Western Europe comprised France, Germany, Italy, Netherlands, Spain, Sweden, United Kingdom. Eastern Europe comprised Bulgaria, Czech Republic, Hungary, Poland, Romania, Russian Federation, Slovakia, Turkey, Ukraine. North America was then taken as the reference region. Results for this analysis are on the following pages.

Table S2. Baseline Characteristics by Region

Characteristic	North America (N=3045)	Central/South America (N=1740)	Asia (N=1602)	Western Europe (N=2373)	Eastern Europe (N=5125)	P-value
Age, median (25th-75th), yrs	67 (61-73)	67 (61-74)	70 (63-75)	67 (60-73)	64 (59-70)	<.001
Female sex, no. (%)	1066 (35.0%)	664 (38.2%)	330 (20.6%)	579 (24.4%)	1249 (24.4%)	<.001
Weight, median (25th-75th), kg	82 (70-94)	74 (65-84)	62 (54-70)	78 (69-88)	80 (70-89)	<.001
Inclusion Criteria for Randomization						<.001
Previous Revascularization, no. (%)	2250 (73.9%)	591 (34.0%)	1162 (72.5%)	1513 (63.8%)	2359 (46.0%)	
ABI value, mean (SD)	0.82 (0.21)	0.70 (0.23)	0.81 (0.25)	0.81 (0.23)	0.72 (0.22)	<.001
ABI or TBI criteria, no. (%)	795 (26.1%)	1149 (66.0%)	440 (27.5%)	860 (36.2%)	2766 (54.0%)	
ABI value, mean (SD)	0.64 (0.12)	0.66 (0.20)	0.62 (0.15)	0.63 (0.12)	0.61 (0.14)	<.001
TBI value, mean (SD)	0.44 (0.16)	0.57 (0.22)	0.46 (0.12)	0.41 (0.16)	0.61 (0.26)	<.001
Limb symptoms, no. (%)						<.001
Asymptomatic	609 (20.0%)	128 (7.4%)	553 (34.5%)	640 (27.0%)	671 (13.1%)	
Mild or moderate claudication	1629 (53.5%)	998 (57.4%)	730 (45.6%)	1239 (52.3%)	2814 (54.9%)	
Severe claudication	690 (22.7%)	508 (29.2%)	212 (13.2%)	427 (18.0%)	1391 (27.1%)	
Pain while at rest	97 (3.2%)	51 (2.9%)	42 (2.6%)	22 (0.9%)	166 (3.2%)	
Minor tissue loss	16 (0.5%)	44 (2.5%)	48 (3.0%)	40 (1.7%)	59 (1.2%)	
Major tissue loss	4 (0.1%)	11 (0.6%)	17 (1.1%)	3 (0.1%)	23 (0.4%)	
Major amputation above the ankle	49 (1.6%)	108 (6.3%)	45 (2.8%)	28 (1.2%)	109 (2.1%)	<.001
Minor amputation	76 (2.5%)	175 (10.1%)	82 (5.1%)	68 (2.9%)	204 (4.0%)	<.001
Medical History, no. (%)						
Stroke	209 (6.9%)	126 (7.2%)	258 (16.1%)	131 (5.5%)	419 (8.2%)	<.001
TIA	191 (6.3%)	42 (2.4%)	62 (3.9%)	86 (3.6%)	126 (2.5%)	<.001
CAD	1437 (47.2%)	449 (25.8%)	308 (19.2%)	588 (24.8%)	1250 (24.4%)	<.001
MI	762 (25.0%)	319 (18.3%)	158 (9.9%)	351 (14.8%)	932 (18.2%)	<.001
Carotid stenosis or carotid revascularization	897 (29.7%)	122 (7.4%)	182 (13.2%)	345 (15.1%)	981 (20.5%)	<.001
Diabetes mellitus type I or II	1220 (40.1%)	989 (56.8%)	689 (43.0%)	779 (32.9%)	1668 (32.5%)	<.001
Hypertension	2627 (86.3%)	1350 (77.6%)	1123 (70.1%)	1792 (75.6%)	3965 (77.4%)	<.001
Hyperlipidemia	2814 (92.4%)	1232 (70.8%)	883 (55.1%)	1942 (81.9%)	3609 (70.4%)	<.001
Tobacco use, no. (%)						<.001
Current	1029 (33.8%)	385 (22.1%)	373 (23.3%)	767 (33.5%)	1735 (33.9%)	
Former	1664 (54.6%)	801 (46.0%)	844 (52.7%)	1254 (54.7%)	1967 (38.4%)	
Never	352 (11.6%)	554 (31.8%)	385 (24.0%)	270 (11.8%)	1423 (27.8%)	
Medication use before randomization, no. (%)						
Aspirin	2327 (76.4%)	1089 (62.6%)	905 (56.5%)	1790 (75.5%)	3160 (61.7%)	<.001
Clopidogrel	1539 (50.5%)	217 (12.5%)	551 (34.4%)	684 (28.8%)	1482 (28.9%)	<.001
Statin	2572 (84.5%)	1134 (65.2%)	1012 (63.2%)	2004 (84.5%)	3459 (67.5%)	<.001
ACE Inhibitor	1381 (45.4%)	706 (40.6%)	205 (12.8%)	998 (42.1%)	2345 (45.8%)	<.001
Angiotensin-receptor blocker	725 (23.8%)	526 (30.2%)	583 (36.4%)	639 (26.9%)	1015 (19.8%)	<.001
Cilostazol	392 (12.9%)	691 (39.7%)	760 (47.4%)	167 (7.0%)	85 (1.7%)	<.001

ABI indicates angle-brachial index; ACE, angiotensin converting enzyme; ARB, angiotensin receptor blocker; CAD, coronary artery disease; MI, myocardial infarction; SD, standard deviation; TBI, toe-brachial index; TIA, transient ischemic attack.

Table S3. Unadjusted Associations Between Region and Clinical Outcomes

	Central/South America			Asia			Western Europe			Eastern Europe			North America	
**Clinical Outcome**	**Incidence Rate (n)**	**HR (95% CI)**	**P-value**	**Incidence Rate (n)**	**HR (95% CI)**	**P-value**	**Incidence Rate (n)**	**HR (95% CI)**	**P-value**	**Incidence Rate (n)**	**HR (95% CI)**	**P-value**	**Incidence Rate (n)**	**Global P-value***
CV death/MI/Stroke	3.87 (151)	0.65 (0.54 - 0.78)	<.001	4.80 (180)	0.80 (0.67 - 0.95)	0.012	3.66 (214)	0.61 (0.52 - 0.72)	<.001	4.09 (510)	0.68 (0.60 - 0.78)	<.001	5.97 (436)	<.001
CV death	2.88 (114)	1.57 (1.23 - 2.01)	<.001	2.23 (87)	1.21 (0.93 - 1.58)	0.163	1.12 (68)	0.61 (0.45 - 0.81)	<.001	2.30 (294)	1.24 (1.02 - 1.52)	0.032	1.85 (143)	<.001
MI	0.66 (26)	0.17 (0.11 - 0.25)	<.001	1.87 (71)	0.68 (0.48 - 0.96)<br>0.33 (0.22 - 0.48)	0.028<br><.001	2.12 (125)	0.53 (0.43 - 0.66)	<.001	1.34 (169)	0.34 (0.28 - 0.41)	<.001	3.96 (292)	<.001
Stroke	0.48 (19)	0.49 (0.30 - 0.81)	0.006	1.44 (55)	1.46 (1.03 - 2.08)	0.032	0.70 (42)	0.72 (0.49 - 1.05)	0.092	0.87 (110)	0.89 (0.66 - 1.20)	0.439	0.97 (74)	<.001
All-cause death	5.05 (203)	1.46 (1.21 - 1.75)	<.001	4.49 (176)	1.28 (1.06 - 1.55)	0.010	2.71 (167)	0.77 (0.64 - 0.93)	0.008	3.43 (440)	0.98 (0.84 - 1.14)	0.774	3.51 (277)	<.001
ALI	0.33 (13)	0.44 (0.24 - 0.80)	0.007	0.60 (23)	0.80 (0.49 - 1.30)	0.372	0.67 (40)	0.91 (0.61 - 1.37)	0.655	0.79 (100)	1.07 (0.77 - 1.49)	0.679	0.74 (56)	0.038
LER	2.49 (95)	0.26 (0.21 - 0.33)	<.001	4.10 (150)	0.44 (0.37 - 0.52)	<.001	8.04 (433)	0.86 (0.76 - 0.98)	0.019	3.53 (429)	0.38 (0.34 - 0.43)	<.001	9.33 (631)	<.001

*Global P-value from the overall association test. Reference is North America. HR (95% CI) and p-values for MI in Asia correspond to time intervals (0,365) and (365,...) days, respectively.
Incidence rate: number of events (n) per 100 patient years.
ALI indicates acute limb ischemia; CI, confidence interval; CV, cardiovascular; HR, hazard ratio; LER, lower extremity revascularization; MI, myocardial infarction.

Table S4. Adjusted Associations Between Region and Clinical Outcomes

Clinical Outcome	Central/South America HR (95% CI)	Central/South America P-value	Asia HR (95% CI)	Asia P-value	Western Europe HR (95% CI)	Western Europe P-value	Eastern Europe HR (95% CI)	Eastern Europe P-value	Global P-value*
CV death/MI/Stroke	0.66 (0.54 - 0.79)	<.001	0.75 (0.63 - 0.89)	0.001	0.65 (0.55 - 0.76)	<.001	0.75 (0.65 - 0.85)	<.001	<.001
Model 1	0.74 (0.61 - 0.90)	0.003	0.86 (0.72 - 1.03)	0.108	0.74 (0.63 - 0.88)	<.001	0.84 (0.73 - 0.96)	0.012	0.003
Model 2	0.69 (0.57 - 0.84)	<.001	0.80 (0.66 - 0.97)	0.020	0.74 (0.62 - 0.87)	<.001	0.84 (0.73 - 0.97)	0.016	<.001
Model 3	0.69 (0.57 - 0.84)	<.001	0.80 (0.66 - 0.97)	0.026	0.75 (0.63 - 0.89)	0.001	0.84 (0.73 - 0.97)	0.018	0.001
CV death	1.53 (1.18 - 1.97)	0.001	1.11 (0.84 - 1.45)	0.466	0.65 (0.49 - 0.87)	0.004	1.37 (1.11 - 1.68)	0.003	<.001
MI	0.18 (0.12 - 0.27)	<.001	0.65 (0.46 - 0.92)<br>0.31 (0.21 - 0.46)	0.015<br><.001	0.57 (0.46 - 0.70)	<.001	0.38 (0.31 - 0.46)	<.001	<.001
Stroke	0.48 (0.29 - 0.81)	0.005	1.34 (0.94 - 1.91)	0.107	0.73 (0.50 - 1.07)	0.103	0.93 (0.69 - 1.27)	0.653	0.001
All-cause death	1.49 (1.23 - 1.79)	<.001	1.16 (0.96 - 1.41)	0.127	0.82 (0.68 - 0.99)	0.044	1.11 (0.95 - 1.30)	0.189	<.001
Model 1	1.55 (1.28 - 1.87)	<.001	1.22 (1.00 - 1.50)	0.047	0.86 (0.71 - 1.05)	0.138	1.14 (0.97 - 1.34)	0.109	<.001
Model 2	1.45 (1.19 - 1.76)	<.001	1.10 (0.89 - 1.35)	0.366	0.81 (0.66 - 1.00)	0.046	1.11 (0.94 - 1.31)	0.238	<.001
Model 3	1.47 (1.21 - 1.79)	<.001	1.15 (0.93 - 1.42)	0.195	0.84 (0.69 - 1.04)	0.103	1.11 (0.94 - 1.31)	0.222	<.001
ALI	0.69 (0.38 - 1.28)	0.238	0.86 (0.52 - 1.40)	0.541	1.04 (0.69 - 1.57)	0.837	1.43 (1.02 - 2.00)	0.039	0.029
LER	0.35 (0.28 - 0.43)	<.001	0.47 (0.39 - 0.56)	<.001	0.96 (0.85 - 1.09)	0.519	0.45 (0.40 - 0.51)	<.001	<.001
Model 1	0.36 (0.29 - 0.45)	<.001	0.48 (0.40 - 0.57)	<.001	0.99 (0.87 - 1.13)	0.885	0.46 (0.41 - 0.53)	<.001	<.001
Model 2	0.36 (0.29 - 0.46)	<.001	0.49 (0.41 - 0.60)	<.001	1.02 (0.90 - 1.16)	0.741	0.48 (0.42 - 0.55)	<.001	<.001
Model 3	0.36 (0.29 - 0.45)	<.001	0.50 (0.41 - 0.61)	<.001	1.03 (0.90 - 1.17)	0.678	0.48 (0.42 - 0.55)	<.001	<.001

*P-value from the overall association test. Reference is North America.
Hazard ratios, 95% confidence intervals, and p-values for MI in Asia correspond to time intervals [0,365] and [365,..] days, respectively.
All outcomes adjusted for age, sex, inclusion criteria, and severity of disease.
Model 1 adjusts for concomitant cardiovascular diseases (prior stroke, carotid stenosis or revascularization, MI, PCI, CABG).
Model 2 adds cardiovascular disease risk factors to Model 1 (diabetes, smoking, hypertension, hyperlipidemia).
Model 3 adds preventive medications to Model 2 (statins, angiotensin-receptive blockers, ACE inhibitors).
HR, hazard ratio; CI, confidence interval; CV, cardiovascular; MI, myocardial infarction; ALI, acute limb ischemia; LER, lower extremity revascularization.

Figure S4. Kaplan Meier event curves for CV death, MI, or ischemic stroke

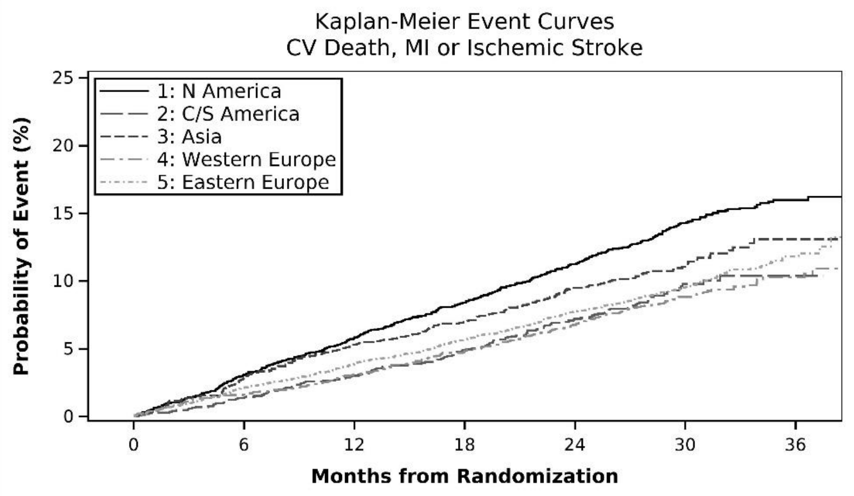

Number at risk:

	0	6	12	18	24	30	36
1	3045	2919	2798	2665	2539	1710	492
2	1740	1680	1615	1563	1500	532	13
3	1602	1535	1481	1435	1370	737	91
4	2373	2290	2234	2176	2099	1363	374
5	5125	4989	4871	4741	4605	2642	417

Figure S5. Kaplan Meier event curves for CV death

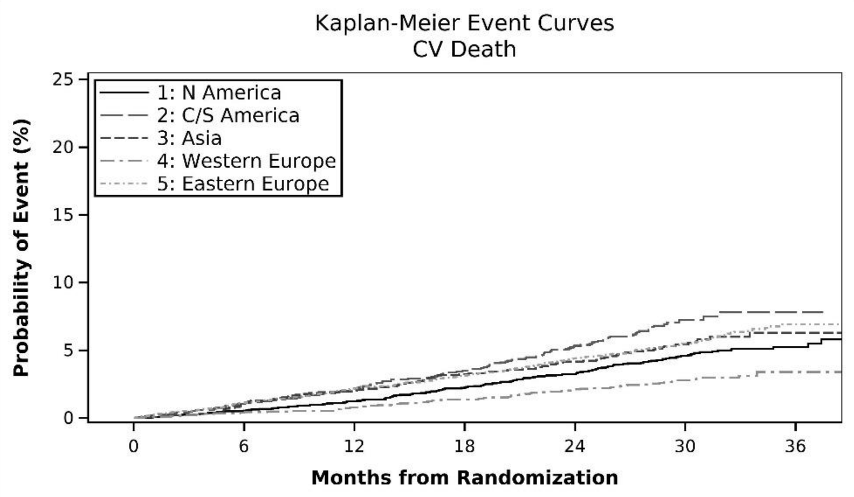

Number at risk:

	0	6	12	18	24	30	36
1	3045	2996	2937	2852	2773	1901	545
2	1740	1689	1633	1594	1538	545	13
3	1602	1565	1534	1498	1456	784	98
4	2373	2328	2300	2268	2221	1456	399
5	5125	5042	4965	4876	4780	2786	449

Figure S6. Kaplan Meier event curves for MI

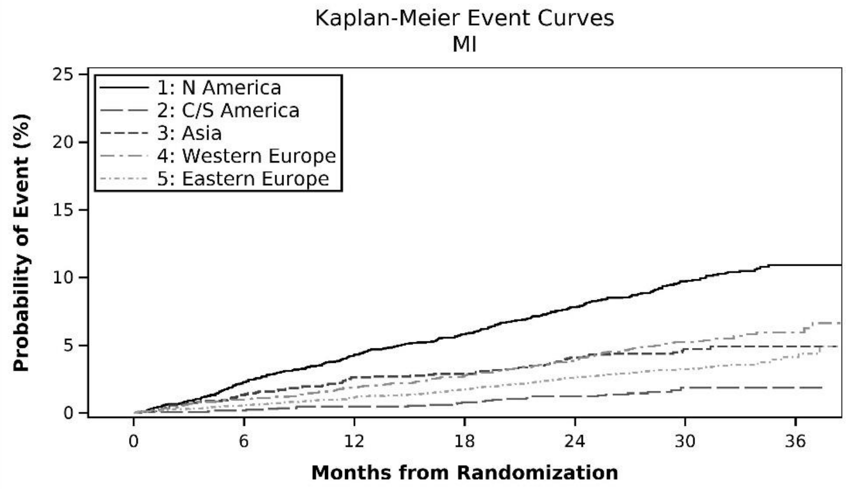

Number at risk:

	0	6	12	18	24	30	36
1	3045	2929	2815	2688	2570	1733	501
2	1740	1680	1620	1572	1511	533	13
3	1602	1544	1497	1458	1402	756	93
4	2373	2298	2244	2197	2124	1378	379
5	5125	5013	4900	4790	4661	2684	428

Figure S7. Kaplan Meier event curves for ischemic stroke

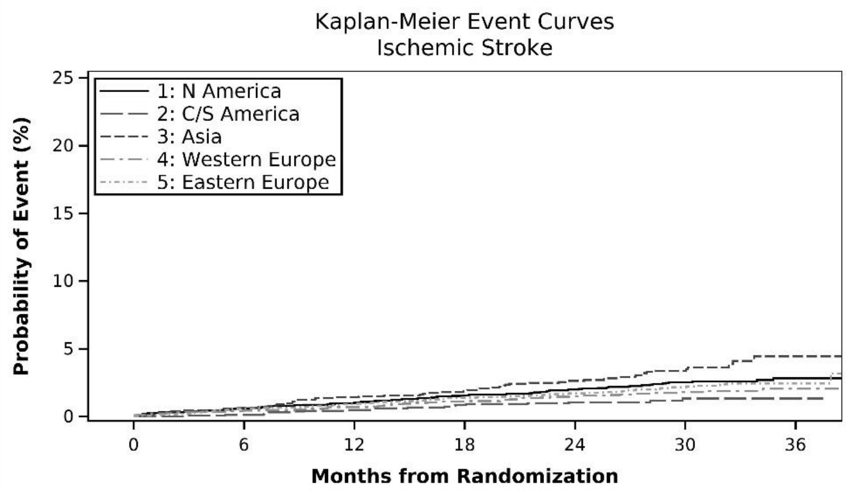

Number at risk:

	0	6	12	18	24	30	36
1	3045	2971	2894	2785	2695	1833	525
2	1740	1680	1621	1571	1515	539	13
3	1602	1552	1512	1465	1411	760	95
4	2373	2307	2266	2224	2161	1416	387
5	5125	5011	4908	4801	4693	2711	429

Figure S8. Kaplan Meier event curves for all-cause death

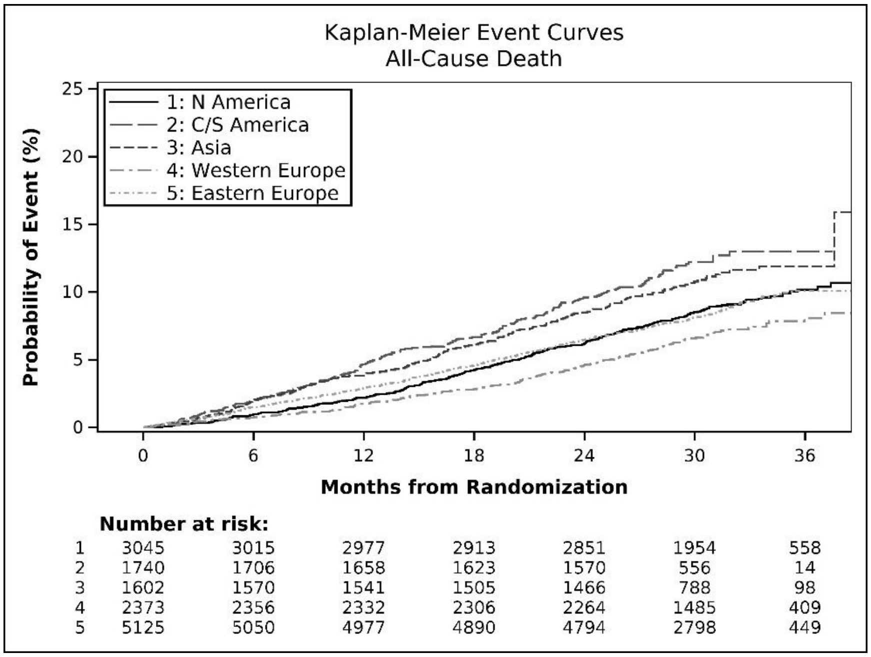

Figure S9. Kaplan Meier event curves for ALI hospitalization

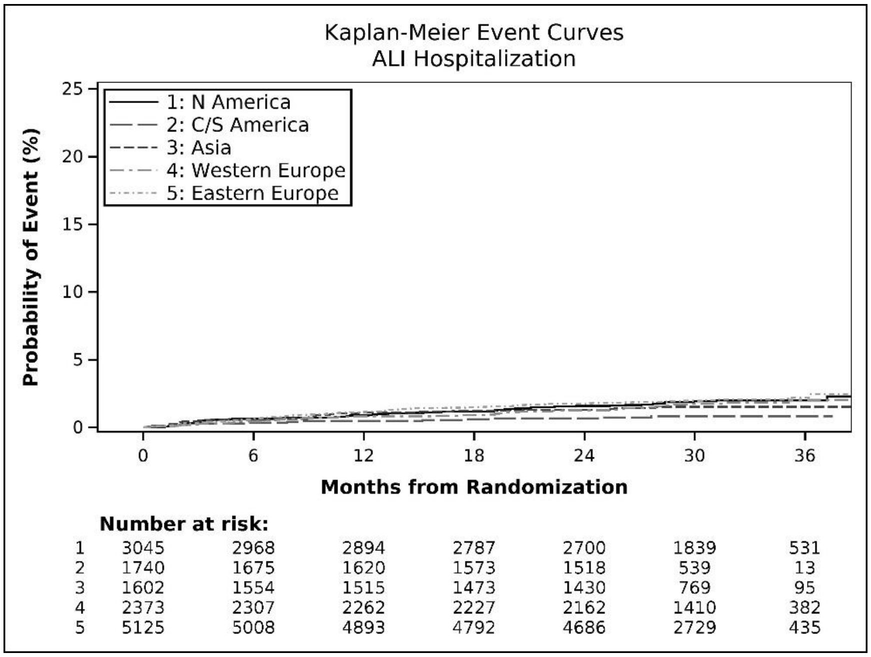

Figure S10. Kaplan Meier event curves for lower extremity revascularization

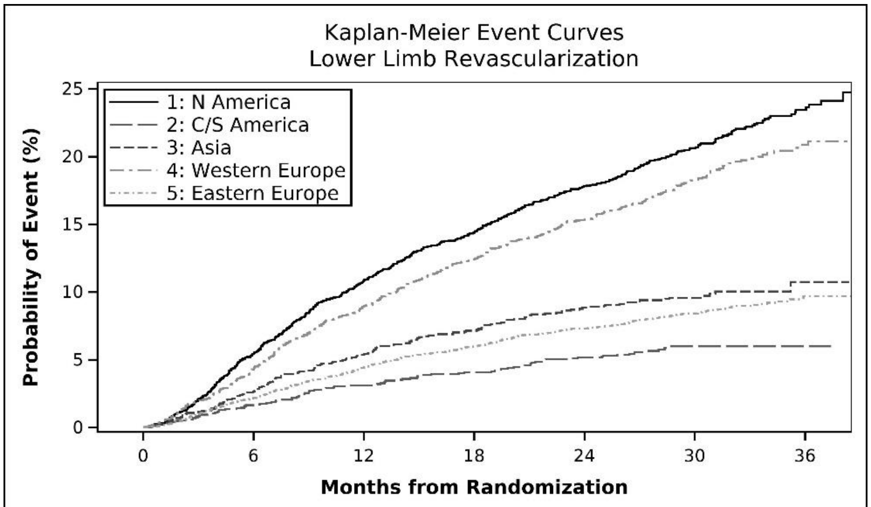

Number at risk:

1	3045	2827	2606	2424	2267	1502	404
2	1740	1653	1579	1520	1452	513	13
3	1602	1523	1450	1387	1325	710	84
4	2373	2220	2078	1973	1860	1166	314
5	5125	4930	4736	4579	4426	2550	406
